# Dynamic Deformation and Perforation of Ellipsoidal Thin Shell Impacted by Flat-Nose Projectile

**DOI:** 10.3390/ma15124124

**Published:** 2022-06-10

**Authors:** Ling Liu, Jianqiao Li

**Affiliations:** State Key Laboratory of Explosion Science and Technology, Beijing Institute of Technology, Beijing 100081, China; 7520170016@bit.edu.cn

**Keywords:** thin ellipsoidal shell, dynamic mechanical behavior, large deformation, lightweight structures, metallic materials

## Abstract

Experimental and theoretical studies were carried out on the dynamic deformation and penetration response characteristics of metal ellipsoidal thin curved shells under impact loads. The deformation characteristics of the impacted ellipsoid shell was investigated via the use of a light gas gun to carry out impact loading experiments at different speeds. Ten cases of experiments were conducted with the impact velocities distributed between 25.69 m/s and 118.97 m/s. Stereo digital image correlation (3D-DIC) technology was applied to capture the dynamic deformation and penetration process of the impacted shell. The recovered shells were measured, and the deformation characteristics were analyzed, along with the dynamic evolution, as observed through 3D-DIC analysis. Based on the experimental results, the displacement mode was summarized and the displacement distribution of the locally impacted ellipsoid shell was proposed. The governing equations were derived for the dynamic deformation and penetration of the impacted ellipsoid shell by means of the Lagrange equation. The proposed theoretical model was verified based on the experimental results. Finally, the influence of the curvature distribution on the impact resistance of ellipsoidal shells is discussed. The results indicated that the proposed theoretical model was effective in analyzing the large deformation and the penetration speed. Stretching the axial length of the ellipsoid shell in the impact direction improved its resistance to penetration. Stretching the axial length of the ellipsoid shell perpendicular to the impact direction improved its resistance to deformation, but reduced its resistance to penetration. Maintaining the triaxial ratio and appropriately reducing the size of the ellipsoidal shell improved its resistance to both deformation and penetration. The above research provides a reference for the analysis of the impact resistance of thin-walled curved shell structures in engineering.

## 1. Introduction

Thin curved shells are widely used in engineering practices such as aerospace, ships, and civil engineering [1]. They are well designed before being applied according to the requirements, except for some unexpected loading, such as impact. This has a significant influence on the safety of thin curved shell structures. A typical impact load for the roofs of large-scale buildings is the impact of a piece of hail during a severe weather event. In extreme weather, hail stones can be very large, and their impact on the curved shell of a roof may generate large deformation and even perforation of the shells of these roofs, which can lead to safety incidents. This kind of unexpected impact load is also a large threat in the field of aerospace engineering, such as impacts by birds, space debris, and spare parts falling from spacecraft. Therefore, it is very important, with a high application value, to investigate local large deformations and perforations of thin curved shells under impact loads during the structural design stage.

Most corresponding investigations have been performed based on numerical simulation and experiments [2,3,4], and it still has been frequently investigated in recent years [5]. Experimental inventions need a good repeatability, which makes the time and economic costs very high. Thus, experiments are always implemented to verify theoretical models and engineering designs. Numerical methods can provide good repeatability and efficiency in the investigation of the dynamic response of locally impacted thin curved shells. However, the accuracy of numerical simulation is governed by the size of the mesh employed. The depressed deformation of the impacted thin curved shell shows a significant local concentration in which the greatest part of the large deformation is limited to a narrow edge region that propagates from the center to the final position of the depressed deformation. Accurate simulation requires either a global fine mesh or an adaptive remesh, according to the evolution of the deformation. Both of these methods will reduce the analysis efficiency and make it difficult to provide efficient feedback for the engineering design. Therefore, it is necessary to develop a theoretical model of the dynamic response of a locally impacted thin curved shell to provide a more efficient, economic, and intuitive reference for the engineering design of curved shell structures. The locally distributed curvature can be represented by an ellipsoidal surface. The reason is that the curved surface can be described by two principle curvatures. The simplest way to describe this curvature distribution is using an ellipsoid with different axial lengths representing different curvature radii. Therefore, a theoretical model for the large deformation of an ellipsoidal shell is necessary in the design of a thin free-curved shell considering the local impact loading applied in engineering.

A spherical shell is a special form of ellipsoidal shell, which has been applied as a representation of a free-curved shell, in addition to direct applications in engineering structures. The reason is that mean Gauss curvature majorly governs the stiffness of curved shells [6]. Therefore, a curved shell could be represented by a spherical shell whose curvature is similar to the mean Gauss curvature of the curved shell. As a typical thin curved shell, spherical shells have been widely investigated in terms of their dynamic responses. In 1972, Updike et al. performed a series of experiments on the dynamic response of a semi-spherical shell under axial compression and drop weight impact loads [7,8,9,10,11,12,13]. The deformation mode and energy absorption properties were obtained. The deformation of the spherical shell was classified into a dimple region and edge region. In 1988, Pogorelov [14] indicated that most of the deformation of a loaded spherical shell could be described by means of an isometric transformation. The deformed part could be approximately considered as the mirror of the original undeformed shell under local impact loading. Based on this assumption of the deformation mode, the small deformation result was solved for an elastic semi-spherical shell loaded by applying a concentrated force on top of the shell. Based on this work, Ning et al. [15] conducted a series of experimental and theoretical investigations on the elastic and plastic deformation of a shallow spherical shell impacted by cylindrical projectiles. A displacement mode was proposed for the edge region with a large deformation concentration, and the influence of the constitutive model was also discussed. Li et al. [16] improved the displacement mode by considering the radial displacement and analyzed the perforation response of the impacted shell. In addition, the influence of parameters in the theoretical model on the deformation and perforation response of the impacted shell was discussed. Recently, several works have focused on the dynamic responses of spherical shells [17,18,19,20] and some composite structures related to spherical shells [1]. However, the curvature of a spherical shell is homogeneously distributed. A spherical shell can be applied as a local approximation of a free-curved shell, but it is unable to show the influence of the curvature on the dynamic response of the curved shell. It is difficult to represent the large local deformation of a free-curved shell using a spherical shell.

Unlike a spherical shell, the curvature is not homogeneously distributed in the case of an ellipsoidal shell. Therefore, an ellipsoidal shell can be used to represent the local geometric properties of a free-curved shell. The local dynamic response of a complex curved shell can be shown by examining the dynamic response of a similar ellipsoidal shell. In addition, an ellipsoidal shell can be directly applied in engineering designs. Therefore, the investigation of the dynamic response of an impacted ellipsoidal shell has more extensive engineering application value. Currently, most investigations of ellipsoidal shells have been performed in the fields of forming, stability, and stress analysis. Bushnell [21] found that the buckling response of the ellipsoidal head used in a pressure container was sensitive to nonlinear geometric properties, which increased the buckling pressure, and nonlinear material properties, which decreased the buckling pressure. Chao [22] explored the analysis of the elastic stress of an ellipsoidal shell with a nozzle under inner pressure. The result was obtained using a graphical solution, according to the stress concentration coefficients of intersections based on dimensionless parameters. Błachut and Jaiswal [23] indicated that defects in ellipsoidal shells had a strong influence on their buckling strength, using a numerical method. The corresponding influence depended on the shape and location of the defect. Smith and Błachut [24] investigated the buckling behaviors of outer-pressure-loaded ellipsoidal shells made of steel by means of experiments and numerical simulations. Magnucki et al. [25] discussed the influence of the thickness and shape of an ellipsoidal head on the stress concentration coefficients of a pressure container design, combining ellipsoidal and cylindrical shell structures. Zheng et al. [26] performed analyses on the buckling properties of large thin ellipsoidal shell heads affected by defects generated during welding. Vella et al. [6] indicated that the stiffness of an ellipsoidal shell depended largely on its mean Gauss curvature, according to their theoretical analysis based on thin-shell theory. Mansoor-Baghaei and Sadegh [27] provided the closed-form solution of an elastic thin ellipsoidal shell impacting an elastic plate. The impact process was divided into two parts—Hertzian deformation from contact and Reissner deformation caused by membrane and bending effects. This was applied to the calculation of parameters such as the maximum compressed deformation, the time, and the impact force. Some of the above investigations were closely related to the dynamic behaviors of ellipsoidal shells. However, few of them focused on the dynamic deformation and failure of impact-loaded ellipsoidal shells. A theoretical model of the large elastic deformation of an impacted ellipsoidal shell would have significant application value in engineering design and would be considered an important reference for structural impact resistance design in relation to complex curved shell structures. Investigations of theoretical models of the dynamic deformation and perforation behaviors of impacted thin ellipsoidal shells are necessary as such models have important applications in engineering design, including aerospace, ships, and civil engineering.

In this study, a series of experimental and theoretical analyses was performed to investigate the large dynamic deformation and perforation behavior of locally impacted thin ellipsoidal aluminum shells. Based on the experimental measurements, the deformation characteristics were determined. This made it possible to describe the displacement distribution in a clear and easy way similar to what was applied to a spherical shell in our previous work [16]. According to the proposed displacement distribution, the strain and curvature distributions were obtained by the local curved coordinates provided by the theory of fields. Based on the Lagrangian equation, the theoretical model was obtained for governing the large deformation of the ellipsoidal shell locally impacted in the normal direction. The theoretical model was validated based on the experimental results. Moreover, the influence of local curvature on the deformation and perforation resistance capability of ellipsoidal shells was discussed. The results of this study could provide theoretical and technical support for a free-curved shell design in protective engineering related to impact.

## 2. Experiments

### 2.1. Specimens and Experimental Method

**Figure 1:**
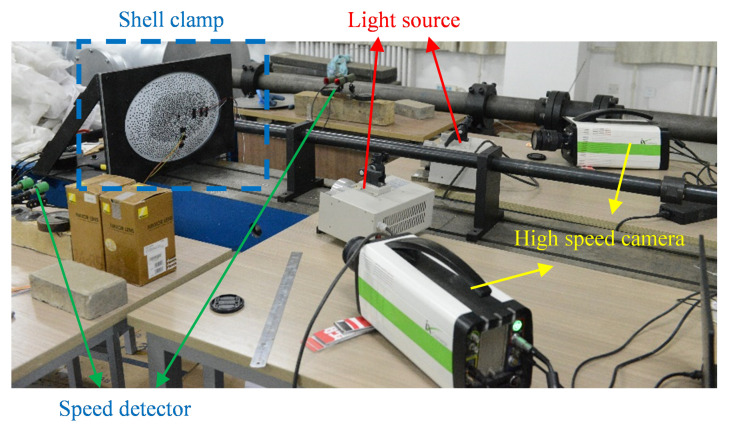
Experimental setup.

**Figure 2:**
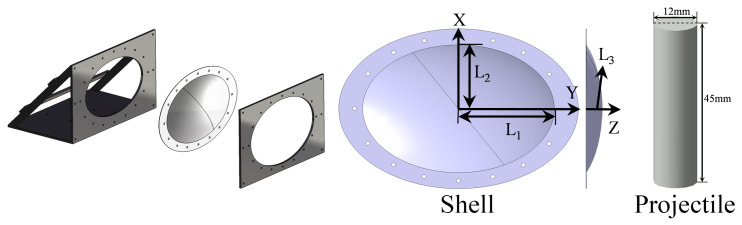
Configuration and sizes of shells.

A light gas gun was applied to launch the projectiles and to perform the impact experiments on the dynamic response of thin ellipsoidal aluminum shells. The diameter of the gun bore was 12.5 mm, and the projectile diameter was 12 mm. Sabot was not used in the test. The highest pressure used in this series of test was 0.9 MPa, and the corresponding launching speed was 118.97 m/s, which was the highest impact speed in this study. A 36.23 m/s impact velocity was achieved by a pressure of 0.1 MPa. A pressure lower than 0.1 MPa sometimes did not work. The velocity of 25.69 m/s was achieved by 0.17 MPa launching, in which the projectile was pushed from the front of the gun to one-quarter length of the gun. The loading and test setup are shown in Figure 1. The fixture of the impacted shell is shown in Figure 2. The boundary condition of the experiment was set as a clamped boundary condition. The edge of the shell was a flat surface of about 40 mm wide, as shown in Figure 2. This part of the shell was fixed between two pieces of 1 cm-thick steel plates by many bolts through the holes in the plates and shells shown in Figure 2. The steel plates were fixed by 6 bolts on the platform connected to the gas gun. This could realize a good clamped boundary condition for the impacted shell. The ellipsoidal shell used in the experiment was the top part of a complete ellipsoid in the axial direction of *c*, which represented the minimum axis of the ellipsoid. The geometric properties of the complete ellipsoid can be described by the relationship between the lengths of all three axes, which were *b*:*a*:*c* = 300 mm:200 mm:100 mm, as applied in the experiments. The ellipsoidal shell, which was a part of the complete ellipsoid, can be described by means of the geometric parameters presented in Figure 2, and the corresponding values are given in Table 1. In addition, the material properties of the ellipsoidal shell are shown in Table 1. The data were given by the material list provided by the company that manufactures the aluminum shells. The projectile used in the experiments was a cylinder made of AISI 1045 steel. The radius and length of all the projectiles were 6 mm and 45 mm, respectively. The projectiles applied in this study were similar to what were used in our previous investigation [16] for shallow spherical shells under impact loads.

**Figure 3:**
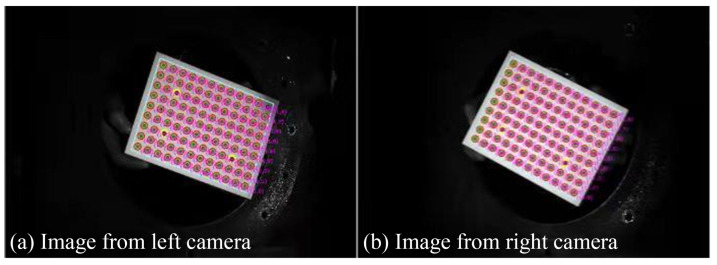
Calibration of DIC measurements.

The MatchID system was used to perform the 3D-DIC measurements for the time-histories of the dynamic response of the impacted ellipsoidal shells and to reconstruct the displacement distribution during the evolution of the ellipsoidal shell deformation. The two cameras used in the DIC measurements needed to be calibrated before the experimental tests, as shown in Figure 3. A grid dot array was applied in the calibration process. A group of figures from the grid dot array in different directions was captured by both cameras, and the camera parameters were obtained via graphic matching and based on the distances between dots on the grid dot array. These graphical parameters were used in the reconstruction provided by the 3D-DIC measurements.

### 2.2. Experimental Results and Analyses

#### 2.2.1. Depressed Deformation Mode and Deformation Boundary Representation

**Figure 4:**
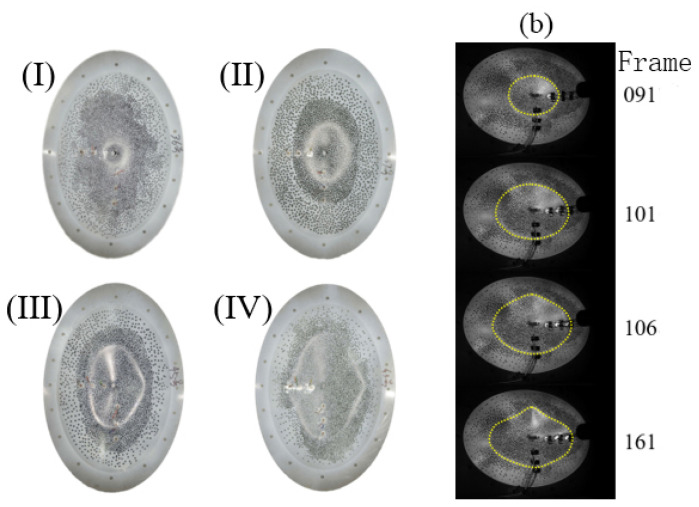
Deformation mode and evolution of impacted ellipsoidal shell. (**I**) for mode I, (**II**) for mode II, (**III**) for mode III and (**IV**) for mode IV. (**b**) indicates the evolution of the deformation mode III.

Four deformation modes were observed on the ellipsoidal shells under local impact loads. According to the shape of the depressed boundary, the deformation modes were classified into four types, including a circular depressed deformation, ellipsoidal depressed deformation, ellipsoidal depressed deformation with buckling in only one direction along the short axis, and ellipsoidal depressed deformation with buckling in two directions along the short axis. These four deformation modes are denoted Mode I, Mode II, Mode III, and Mode IV, respectively, as shown in Figure 4.

**Figure 5:**
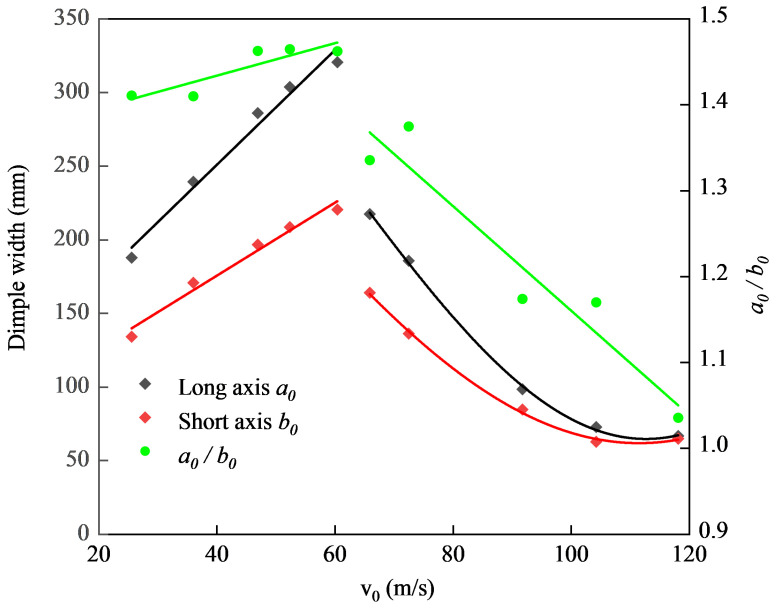
Dual axes of the dimple boundary that changed with impact velocities.

The results presented in Table 2 showed that when the deformation was small, the depressed deformation boundary of the ellipsoidal shell was elliptical Mode I without buckling deformation. This was indicated by the deformation mode for high-velocity impact. The deformation of the lowest velocity impact in the test was Mode II. An impact at a lower velocity was not performed because it was very difficult for the utilized light gas gun with almost a 10 m length. However, the impact at a very low velocity would generate deformation Mode I because of the very small deformation generated by the low-velocity impact. With the increase in the impact velocity, the deformation developed from Mode I to Mode II, and with the gradual increase in the impact velocity, the depressed deformation area gradually increased and the deformation mode further developed into deformation Mode III, with bilateral buckling deformation along the short axis. When the impact velocity further increased, the ellipsoidal shell was penetrated at a critical velocity of 60.78 m/s–66.32 m/s. As shown in Figure 5, the deformation mode of the ellipsoidal shell changed to Mode III after penetration, the depressed deformation area decreased sharply with an increase in the impact velocity, and the deformation mode changed to Mode I through Mode II.

Figure 4b shows the development process of deformation Mode III. In the initial stage of the impact load, the depression deformation was actually Mode II and remained Mode II throughout the entire impact load process, that is the boundary of the deformation area was elliptical. In the end stage of deformation development, the shape of the depression deformation boundary suddenly lost stability, resulting in buckling deformation on the minor axis side, and finally evolved into deformation Mode III. Deformation Mode IV underwent a similar development process to that of deformation Mode III. However, for deformation Mode I, there was no degradation process from an ellipse to a circle, and the deformation region boundary of the approximate circle was maintained throughout the entire deformation development process.

The results of the static analysis and dynamic analysis of high-speed photography showed that under the impact loads, only two deformation modes were observed in the impacted ellipsoidal shell in the deformation development stage, namely the approximate circular deformation (Mode I) in the small deformation stage and the elliptical deformation (Mode II) in the continuous development process of the deformation. Although buckling instability occurred in the case of the large deformation that generated deformation Modes III and IV, the high-speed photography results of the deformation development process showed that these two modes only changed the shape of depressed deformation and had no significant influence on the size of the deformation area. When considering the impact resistance of thin shell structures, the most important factors are the dimple depth, dimple size, and ballistic limit of the thin shell under impact loading. The dimple size is represented by the dimple width. The boundary of the depressed deflection was an ellipse, and the size of this dimple deformation could be described by the long and short axes of the elliptical boundary of the depressed deflection. Normally, dimple width is defined as the short axis of the elliptical defect boundary. If the long axis is discussed at the same time, a label is provided to mark each kind of axis. The largest deflection displacement that occurred during the deformation was the deflection of the center of the shell. The corresponding displacement was defined as the dimple depth. The buckling deformation of the ellipsoidal shell had no considerable influence on the depth or size of the depressed deformation. In addition, the main buckling came from the minor axis direction, and the additional deformation caused by buckling was not prominent relative to the minor axis of the deformation area, which had little impact on the geometric characteristics. This introduced great convenience into the geometric description of the dynamic deformation of the ellipsoidal shell under the impact load. In the theoretical analysis, it was not necessary to consider deformation Modes III and IV. Moreover, the circular deformation boundary was considered as a special case of the elliptical deformation boundary, which could thus be described by means of the elliptical deformation characteristics. Therefore, the dynamic deformation boundary characteristics of the ellipsoidal shell under local impact load could be fully characterized by deformation Mode II.

On this basis, all the depressed deformation modes were approximated as ellipses, and the characteristics of the major axis and minor axis of the depressed deformation boundary were analyzed. The measurements were carried out for the static recovered test piece, and the results are shown in Figure 5.

As shown in Figure 5, the results indicated that the ballistic limit of the locally impacted ellipsoidal shell was between 60.78 m/s and 66.32 m/s. After being perforated, the depressed deformation area of the ellipsoidal shell decreased sharply with the increase in impact velocity. The results in Figure 5 show that before the ellipsoidal shell was perforated, the ratio of the major axis to the minor axis of the ellipse of the depressed deformation boundary was between 1.4 and 1.5, which was very close to the ratio of the major axis to the minor axis of the original ellipsoidal shell. After the shell was perforated, with the increase in impact velocity, the size of the dimple area decreased rapidly. At the same time, the proportion of the long and short axes of the ellipse at the depressed deformation boundary decreased rapidly from 1.5 to 1.0, which meant that the shape of the dimple boundary changed rapidly from an ellipse into a circle. In terms of the deformation characteristics, the ratio of the major and minor axes of the ellipse of the dimple boundary changed linearly with respect to the impact velocity. Because the depressed deformation of the ellipsoidal shell decreased rapidly after penetration, the residual velocity of the projectile was more important than the depressed deformation characteristics for the impact resistance characteristics of the ellipsoidal shell. Before penetration, the deformation characteristics of the ellipsoidal shell played the most important roles in its impact resistance characteristics. The experimental results showed that in the stage of significant deformation, the parameters of the ellipse of the deformation boundary demonstrated outstanding characteristics and the ratio of its major axis to its minor axis was very close to the biaxial ratio of the original ellipsoidal shell. With the increase in deformation, the ellipse of the depression boundary approached the contour ellipse of the original ellipsoidal shell, which was a very important feature, indicating that, in the process of large deformation depression and its development, the depressed deformation boundary was almost in the same plane, and the ellipse of the depression boundary had the same proportion of the major axis and the minor axis as the ellipse of the ascending surface of the original ellipsoidal shell. This expressed a very specific relationship of deformation characteristic parameters, which was used for establishing the corresponding theoretical analysis model.

#### 2.2.2. Characterization and Representation of Dimple Depth

**Figure 6:**
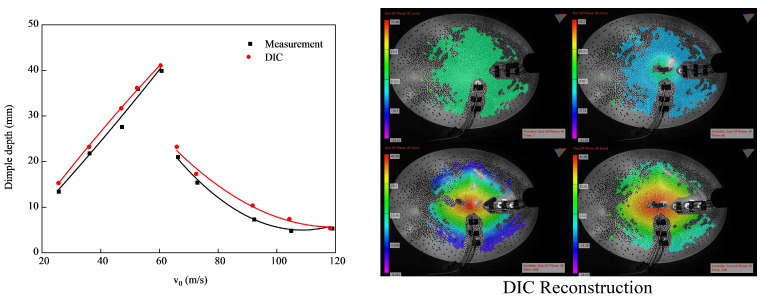
Comparison between dimple depth captured via DIC and via the static measurement of recovered specimens, which changed with impact velocities.

Due to the obvious characteristics of the impact point on the ellipsoidal shell, it was easy to statically measure the final deformation depth. However, it was difficult to obtain the distribution of the dimple depth along a certain direction in order to statically measure the recovered specimen. Manual measurement would introduce many uncertainties. If the specimen was cut, it might cause the release of the residual deformation, and the measured result would not show the depth distribution of the final deformed specimen. The distribution of the dimple depth could be determined, on the other hand, by reconstructing the deformed surface using the DIC method. The reliability of the results obtained from the DIC test was verified by comparing the statically measured dimple depth with the DIC results, and this comparison is shown in Figure 6. The measured data were obtained after releasing the specimens from the fixtures. The constraint provided by the hoop strain had a slight release, which generated a tiny release of the constrained elastic deformation. It was considered as the reason why almost all DIC results were greater than the measured data, as shown in Figure 6. The average difference between the measurement results of the two measurement methods was 1.474 mm. This indicated that reliable dimple depth distribution results could be obtained through the DIC testing.

**Figure 7:**
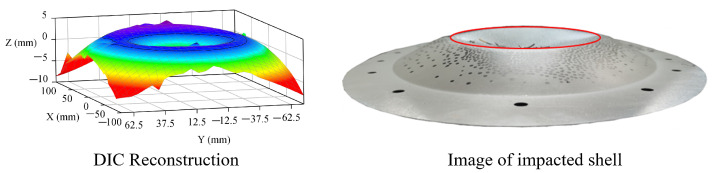
Boundary shape of the depressed deformation.

The DIC reconstructions of the experimental results are shown in the right-hand image in Figure 6. Due to the influence of the curvature of the curved shell and the trajectory of the projectile, the reconstruction of the displacement results exhibited many discontinuous positions in the long-axis direction at different times in the impact process. Therefore, the short-axis direction was selected to consider the displacement distribution measured using the DIC. Combined with the evolution results of the depressed deformation boundary in the impact process, the dimple boundary was located in the same plane before buckling, and the dimple elliptical boundary had a curvature very close to that of the original ellipsoidal shell. This can be observed in Figure 7. That meant that the dimple depth distribution in the long-axis direction was similar to that in the short-axis direction. The dimple depth distribution in the long-axis direction could be obtained through analyzing the dimple depth distribution in the short-axis direction.

**Figure 8:**
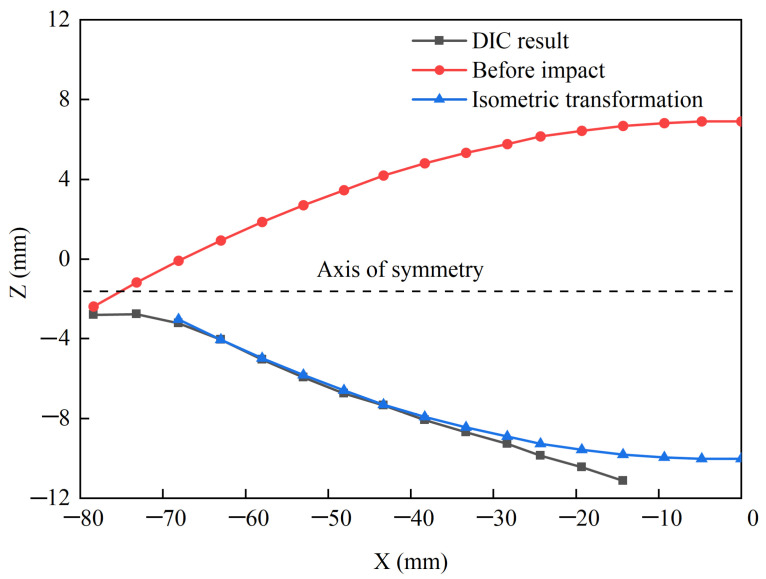
Validation of isometric transformation.

Figure 8 shows the dimple depth distribution results in the minor-axis direction obtained via DIC testing when the length of the minor semi-axis in the dimple area was approximately 80 mm. In Figure 8, the section shape of the minor axis of the original ellipsoidal shell and its mirror flip shape were observed at the same time. The comparison between the DIC test results and the mirror flip shape showed that the actual deformation did not conform to the mirror transformation in the range of 0 to 30 mm, whereas the mirror transformation showed good agreement with the actual deformation in the range of 30 to 80 mm. That is, in most deformation areas, the dimple depth could be described by referring to the mirror transformation of the original ellipsoidal shell, whereas for the small central area, the dimple depth was almost linearly distributed in the minor-axis direction. In a narrow area near the deformation boundary of the depression, the displacement distribution had no significant characteristics.

According to the static measurement of the recovered specimens and the dynamic DIC test results of the impacted ellipsoidal shell, the geometric characteristics of the depressed deformation were obtained for the ellipsoidal shell under local dynamic impact loading. In the case of large depressed deformation, the boundary of the deformation area was almost in the same plane, and the shape of the dimple boundary was an oval. The long and short axes of the elliptical dimple boundary were in the same proportion as the long and short axes in the original ellipsoidal shell surface. In most areas of large deformation, the distribution of dimple depth was the result of the mirror transformation of the original ellipsoid. In a small part of the central area, the dimple depth was linearly distributed. This is summarized in Figure 8 in which the DIC results in the range of [−40,0] were almost linearly distributed. The results did not include data in the range of [−15,0] because it was covered by the projectile at the given time. There was a narrow edge area at the depressed deformation boundary. The displacement of the edge area changed significantly; however, it is not easily described as Region 2, and the displacement cannot be directly obtained from the images. The experimental methodology included both the final measurement of the recovered specimens and the dynamic evolution of the deformation. The characteristics of the deformation were obtained including both spatial distribution and time evolution. A very clear deformation description was captured by the proposed experimental methodology, which provided a very strong foundation for establishing the theoretical model. The experimental results showed that the depressed deformation of the ellipsoidal shell under local impact load had similar response characteristics to the spherical shell, which provided a good geometric characteristic support for establishing the theoretical model of the dynamic response of an impacted ellipsoidal shell. In the theoretical analysis, an isometric transformation method similar to that of the spherical shell was used to determine the displacement, strain, and curvature distribution of the ellipsoidal shell, which provided a foundation for the establishment of the theoretical model.

## 3. Theoretical Investigation

In our previous study [16], a theoretical model was proposed for spherical shells. The deformation mode in the spherical shell was very simple. If the buckling was ignored, the only deformation mode was a circular dimple. Compared with the deformation of the spherical shell, the ellipsoidal shell was much more complex. Fortunately, an important assumption applied in the previous studies of spherical shells was available form the ellipsoidal shell. This was proven by experiments performed in this paper. The first one was that mirror transformation could also be used for ellipsoidal s for a large central part of deflection. The second one was that the dimple boundary was in the same iso-surface along the impact direction and only displacement along the impact direction was considered. This ensured the dimple shape and gave a method to describe the dimple deformation. Based on the affine transformation, the ellipsoidal shell could be transformed to a spherical shell. The elliptical dimple boundary could also be transformed to a circular dimple boundary similar to the spherical shell. Our previous work provided good references for the work conducted in this paper. However, there were still some issues in need of solving. The most important one was the way to calculate the strain and curvature of the deformed shell.

### 3.1. Displacement Model of Ellipsoidal Shell

**Figure 9:**
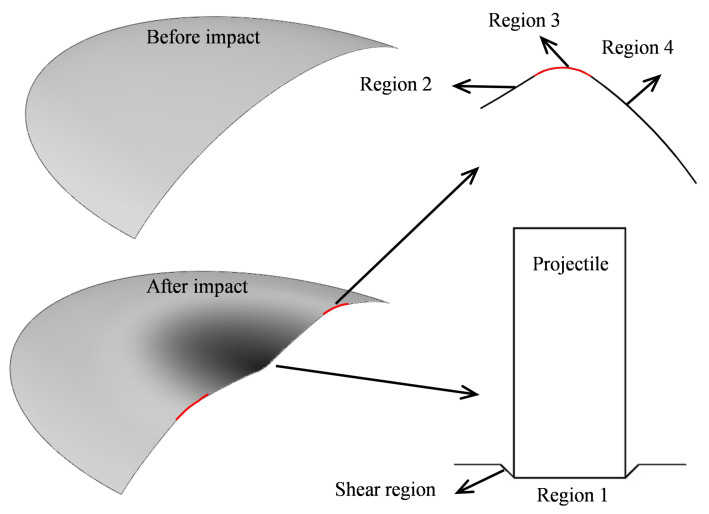
Deformation features of an impacted ellipsoidal shell.

Based on the local dynamic normal impact load experiments using ellipsoidal thin shells, the geometric characteristics of the depressed deformation boundary and surface were measured, and the depressed deformation mode was obtained. Under the local normal impact, the depressed deformation of an ellipsoidal thin shell had distinct characteristics. Most areas of the depressed deformation had a good mirror transformation relationship with the original surface, and only a small part of the depressed deformation surface in the central area had a linear distribution after deformation. In this section, the theoretical model of dynamic deformation of an ellipsoidal thin shell under local normal impact was established based on the energy principle, with particular attention to the dynamic deformation of an ellipsoidal thin shell. In this case, for the final state, the energy involved in a small part of the central area was relatively low, considering that of the entire deformed shell. When the local deformation was small, the kinetic energy of the impact load was dominant for the dynamic evolution process. The kinetic energy of the non-mirror deformation in the central area was not important because of its rather low value compared with the overall energy of the deformed shell. Therefore, an assumption was made for the theoretical model of the dynamic deformation process of an ellipsoidal thin shell that the deformation mode of other areas in the depressed deformation area were equivalent to the mirror transformation of the original surface, except for the edge area. In addition, the edge of the central impact area was considered a shear plug deformation, and shear plug failure and penetration were considered in the final fracture calculations. Furthermore, only the displacement in the impact loading direction was considered, and the displacement in all other directions was ignored. Thus, the deformation mode and displacement distribution of each region were determined. As shown in Figure 9, the deformation of an ellipsoidal thin shell after impact was divided into four regions: Region 1 was the impact region; Region 2 was the mirror depression region; Region 3 was the edge region; Region 4 was the undeformed region.

The displacement of Region 1 is represented by $w_{1}=\gamma$. According to isometric transformation, the shape of Region 2 was symmetrical to the original shell on the plane $z=z_{f}$. Considering a rigid displacement $z_{h}$ led by the inner boundary between Region 2 and Region 3, the displacement distribution of Region 2 of an impacted ellipsoidal shell was shown as $w_{2}=z_{h}+2\left(z-z_{f}\right)$. The displacement $w_{3}$ in Region 3 was assumed to be a second-order polynomial distribution in the impact direction. This distribution was solved by zero- and first-order continuous conditions that needed to be satisfied in both of the two boundaries of Region 3, that is the inner boundary between Regions 2 and 3 and the outer boundary between Regions 3 and 4. The solved displacement distribution of Region 3 is shown in Equation (1), combined with the displacement of Regions 1 and 2.

(1) $$w_{1}=\gamma ;\;\;w_{2}=z_{h}+2\left(z-z_{f}\right);\;\;w_{3}=\frac{{\left(z-z_{f}+z_{h}\right)}^{2}}{z_{h}}$$

Considering only the displacement in the impact load direction, i.e., axial displacement, the deformed configuration can be easily described based on the original curved surface configuration. Taking the impact direction as the *z* direction, i.e., the height direction of the ellipsoidal thin shell, all the height descriptions were under the configuration of the undeformed ellipsoidal thin shell, i.e., the height $z_{f}$ of the boundary of Region 2 corresponding to the original ellipsoidal surface, which was the height of any point on the deformed ellipsoidal surface on the original ellipsoidal surface. The height difference between the inner and outer boundaries of Region 3 on the original surface was a parameter to be determined in the displacement distribution model.

Region 4 was always contained in the displacement mode. That was an undeformed part of shell, and the boundary displacement was zero, which represented a clamped constraint. Therefore, an explicit boundary condition was not necessary in the theoretical model, where the clamped constraint was implied.

### 3.2. Distribution of Curvature and Strain

Since there was no displacement and deformation in Region 4, only the deformation distribution of Regions 1, 2, and 3 needed to be considered. The deformation of Region 1 was considered a shear plug deformation, which was concentrated on the interface between Regions 1 and 2. According to the displacement distribution, Regions 1 and 2 were discontinuous and could not describe the shear deformation. Therefore, it was assumed that a shear deformation region with limited width existed between Regions 1 and 2. The region was a circular ring; the radial width of the ring was δr, and the displacement was linearly distributed in the width, which was consistent with the assumptions used for the analysis of shallow spherical shells given in the literature [16]. The shear strain of the shear plug deformation was similar to that given in the literature [16] and can be shown as follows.

(2) $$\epsilon_{\tau }=\frac{\gamma -w_{2}\left(x=r_{p},y=0\right)}{2\delta r};\;\;{\dot{\epsilon }}_{\tau }=\frac{\dot{\gamma }-{\dot{w}}_{2}\left(x=r_{p},y=0\right)}{2\delta r}$$

where $\epsilon_{\tau }$ and ${\dot{\epsilon }}_{\tau }$ are the shear strain and shear strain rate of Region 1. $r_{p}$ is the radius of the projectile. The displacement of point $x=r_{p},y=0$ was selected to represent the displacement and velocity of the outer boundary between Region 1 and Region 2 to give the displacement and velocity gradient between Regions 1 and 2.

Region 2 was an isometric transformation of the original shell, and the corresponding membrane forces were 0, which was different from edge Region 3. In addition, both Region 2 and Region 3 exhibited curvature deformation, and the curvature of thin shell can be calculated based on geometric equations, as expressed in Equation (3) [28].

(3) $$\begin{array}{cc} k_{1} & =-\frac{1}{A_{1}}\frac{\partial }{\partial \xi }\left(\frac{1}{A_{1}}\frac{\partial w}{\partial \xi }\right)-\frac{1}{A_{1}A_{2}}\frac{\partial A_{1}}{\partial \eta }\left(\frac{1}{A_{2}}\frac{\partial w}{\partial \eta }\right)\\ k_{2} & =-\frac{1}{A_{2}}\frac{\partial }{\partial \eta }\left(\frac{1}{A_{2}}\frac{\partial w}{\partial \eta }\right)-\frac{1}{A_{1}A_{2}}\frac{\partial A_{2}}{\partial \xi }\left(\frac{1}{A_{1}}\frac{\partial w}{\partial \xi }\right)\\ k_{12} & =-\frac{1}{A_{1}A_{2}}\left(\frac{\partial^{2}w}{\partial \xi \partial \eta }-\frac{1}{A_{1}}\frac{\partial A_{1}}{\partial \eta }\frac{\partial w}{\partial \xi }-\frac{1}{A_{2}}\frac{\partial A_{2}}{\partial \xi }\frac{\partial w}{\partial \eta }\right) \end{array}$$

where $A_{i}\left(i=1,2\right)$ represents Lame’s coefficients. *w* indicates displacement along the normal direction. The details in the calculation of the above curvatures is given in Appendix A. In addition, the strain distribution is also provided in Appendix A. Based on the curvatures and strains derived in Appendix A, the general forces involved in the following section of governing equations can be expressed.

### 3.3. Governing Equations

Based on Hamilton’s principle, the governing equations of the dynamic deformation and the perforation of an impacted ellipsoidal shell could be given by the Lagrangian equations.

(4) $$\begin{array}{cc} \frac{\partial^{2}T}{\partial {\dot{\chi }}^{2}}\ddot{\chi }+\frac{\partial^{2}T}{\partial \dot{\chi }\partial \chi }\dot{\chi } & =\frac{\partial T}{\partial \chi }-Q_{\chi }\\ \frac{\partial^{2}T}{\partial {\dot{\gamma }}^{2}}\ddot{\gamma }+\frac{\partial^{2}T}{\partial \dot{\gamma }\partial \gamma }\dot{\gamma } & =\frac{\partial T}{\partial \gamma }-Q_{\gamma } \end{array}$$

where χ,γ are the dimple radius along the short axis and the displacement of Region 1, respectively. *T* is the kinetic energy of the system. $Q_{i}\left(i=\chi ,\gamma \right)$ is the general force. The kinetic energy of the system can be integrated from the shell based on the displacement distributions.

(5) $$T=\frac{1}{2}\rho h\int \int_{S}{\dot{w}}^{2}dS$$

where ρ and *h* are the density and thickness of the shell, and this remained constant during the deformation. *S* is the area of the depressed part of the deformed shell. The kinetic energy *T* is integrated around all regions, including Regions 1, 2, and 3. The governing equation depended on the derivation of the kinetic energy, rather than the kinetic energy *T*. To obtain the expression of the derivation of the kinetic energy, some simplifications were needed for the ellipsoidal equation.

(6) $$\begin{array}{cc} z & =c\sqrt{\left(1-\frac{x^{2}}{a^{2}}-\frac{y^{2}}{b^{2}}\right)}\approx c\left[1-\frac{1}{2}\left(\frac{x^{2}}{a^{2}}+\frac{y^{2}}{b^{2}}\right)\right]\\ z_{f} & =c\sqrt{\left(1-\frac{\chi^{2}}{a^{2}}\right)}\approx c\left[1-\frac{1}{2}\frac{\chi^{2}}{a^{2}}\right] \end{array}$$

According to Equation (6), the velocity distributions of each region of the deformed shell were simplified as follows:

(7) $$\begin{array}{cc} {\dot{w}}_{1} & =\dot{\gamma }\\ {\dot{w}}_{2} & =\frac{2c\chi \dot{\chi }}{a^{2}}\\ {\dot{w}}_{3} & =\frac{c\chi \dot{\chi }\left(\chi^{2}b^{2}c^{2}+2a^{2}b^{2}\epsilon -a^{c}y^{2}-b^{2}cx^{2}\right)}{a^{4}b^{2}z_{h}} \end{array}$$

Substituting Equation (7) into Equation (5), the derivations of the kinetic energy are given by Equation (8).

(8) $$\begin{array}{cc} \frac{\partial^{2}T}{\partial {\dot{\chi }}^{2}} & =\frac{\chi^{2}\left(\frac{3}{64}A+\frac{3}{8}B+\frac{3}{2}C+\frac{3}{2}D+E\right)}{F}\\ \frac{\partial T}{\partial \chi } & =\frac{\chi {\dot{\chi }}^{2}\left(\frac{3}{16}A+\frac{9}{8}B+\frac{3}{2}C+3D+E\right)}{F}\\ \frac{\partial^{2}T}{\partial \dot{\chi }\partial \chi } & =\frac{2\chi \dot{\chi }\left(\frac{3}{16}A+\frac{9}{8}B+\frac{3}{2}C+3D+E\right)}{F}\\ \frac{\partial^{2}T}{\partial {\dot{\gamma }}^{2}} & =M_{p}+\pi \rho hr_{p}^{2} \end{array}$$

where

(9) $$\begin{array}{cc} A & =-c^{3}\left(a^{2}\kappa^{2}+3ab\kappa +\frac{8}{3}b^{2}\right){\left(a\kappa -b\right)}^{3}\chi^{6}\\ B & =a^{2}b^{2}c^{2}z_{h}\left(a\kappa +2b\right){\left(a\kappa -b\right)}^{2}\chi^{4}\\ C & =-a^{5}b^{4}cz_{h}^{2}r_{p}^{2}\\ D & =a^{4}b^{5}c\chi^{2}\\ E & =a^{6}b^{5}z_{h}^{3}\\ F & =\frac{8\pi c\rho h}{3a^{9}b^{4}z_{h}^{2}} \end{array}$$

κ represents the axial ratio of the ellipsoid describing the boundary of the depressed deformation. $M_{p}$ represents the projectile mass.

**Figure 10:**
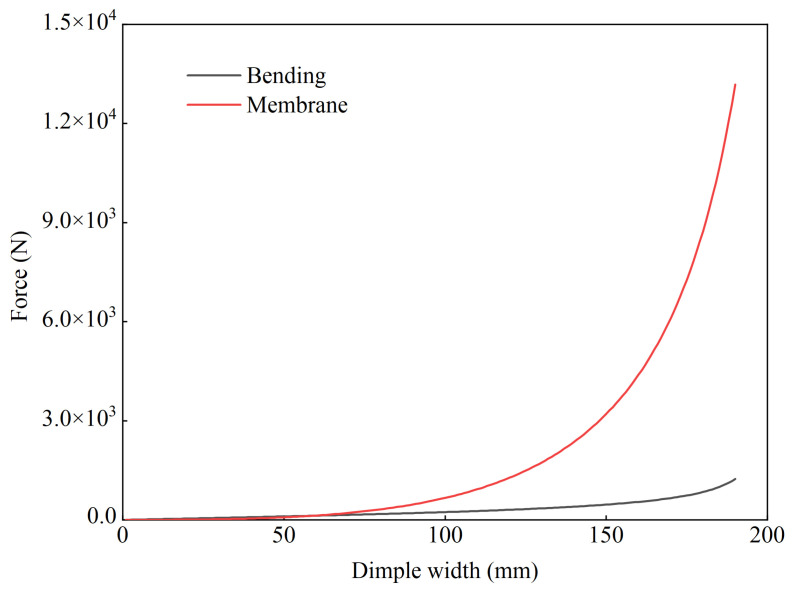
Comparison between general force $Q_{\chi (3)b}$ and $Q_{\chi (3)m}$.

The corresponding general forces in Equation (4) were obtained as shown in Equation (10).

(10) $$\begin{array}{cc} Q_{\chi } & =Q_{\chi (1)}+Q_{\chi (2)}+Q_{\chi (3)}\\ Q_{\gamma } & =-2\pi h\tau \delta r\left(2r_{p}+\delta r\right)\left(1-\frac{\gamma -w_{2}{|}_{x=r_{p},y=0}}{h}\right)\frac{\partial \epsilon_{\tau }}{\partial \chi }\\ Q_{\chi (1)} & =-Q_{\gamma }\\ Q_{\chi (2)} & =0\\ Q_{\chi (3)} & =Q_{\chi (3)b}+Q_{\chi (3)m} \end{array}$$

where $Q_{\chi (2)}=0$, because Region 2 was an isometric transformation of the original undeformed shell and the corresponding strain was zero. The curvature of Region 2 was independent of the time evolution of the deformation. $Q_{\gamma }$ and $Q_{\chi (1)}$, as well as the shear stress τ were provided in [16]. $Q_{\chi (3)b}$ represents the contribution from the bending moment, and $Q_{\chi (3)m}$ represents the contribution from the membrane stress. The expression of the bending curvature was complex. The general force $Q_{\chi (3)b}$ was compared with $Q_{\chi (3)m}$ for different dimple widths, and the results are shown in Figure 10.

The comparison indicated that general force $Q_{\chi (3)}$ was mainly determined by the membrane stress contribution $Q_{\chi (3)m}$ rather than the bending moment contribution $Q_{\chi (3)b}$. This meant that $Q_{\chi (3)b}$ could be ignored. The general force $Q_{\chi (3)}$ is expressed as follows:

(11) $$Q_{\chi (3)}=Q_{\chi (3)m}=\frac{2b\sigma_{0}hc^{2}\chi }{z_{f}}\int_{z_{f}-z_{h}}^{z_{f}}\int_{0}^{2\pi }\frac{(2\left(z-z_{f}\right)+z_{h})G}{\left(a^{2}z^{2}+c^{2}G\right)z_{h}}zd\theta dz$$

where $\sigma_{0}$ is the static yield stress of the shell material. θ and *G* is defined in Appendix A.

In the calculation of the theoretical model, the following initial conditions were applied, according to the experiments.

(12) $$\begin{array}{cc} {\chi |}_{t=0} & =r_{p},\;\;\dot{\chi }{|}_{t=0}=0;\\ {\gamma |}_{t=0} & =\frac{a^{2}h+c\left(\chi^{2}-r_{p}^{2}\right)}{a^{2}},\;\;\dot{\chi }{|}_{t=0}=\sqrt{\frac{M_{p}v_{0}^{2}}{M_{p}v_{0}^{2}+\pi \rho hr_{p}^{2}}}; \end{array}$$

where *t* is the solving time and $v_{0}$ is the impact velocity of the projectile. $r_{p}$ is the radius of the projectile. The initial velocity was obtained considering energy conservation by ignoring the initial deformed energy.

## 4. Analysis of Theoretical Results and Discussion

### 4.1. Validation of the Theoretical Model

**Figure 11:**
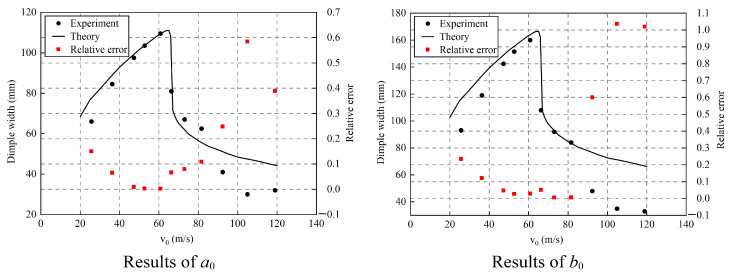
Validation of theoretical model via comparison with the experimental results..

The results shown in Figure 11 indicated that when the depressed deformation was large, the theoretical results were in good agreement with the experimental results. In our previous investigation on spherical shells impacted by flat-nose projectiles [16], the theoretical predictions on dimple width agreed very well with the experimental results for all speeds. However, the comparison of dimple width between experimental and theoretical results indicated that the theoretical predictions agree well with experiments only for those results given by a high speed that is smaller than the ballistic limit. That means only the predictions of dimple width for large deformation agreed well with the experimental results. The reason is that deformation Mode II is only appropriate for large deformation, and this deformation mode is the basic assumption made in the theoretical model. It is known, based on the experimental results, that the depressed deformation area of the ellipsoidal shell was approximately an ellipse with a ratio between the major and minor axes that was similar to that of the original ellipsoidal shell. This conformed to the isometric transformation assumption used in the theoretical model presented in this paper. Therefore, the theoretical results in the large deformation range were in good agreement with the experimental results. The relative errors of the predictions for both the short axis and long axis were less than 5%, as shown in Figure 11. For the case of a small depressed deformation, it is known from the experiments that the proportion of major and minor axes in the dimple area gradually decreased with the reduction of the deformation area. Therefore, the theoretical results in this study involved a level of uncertainty for the case of small depressed deformation, especially for velocities larger than the ballistic limit. The relative errors of impacts at low speeds were less than 15% for the short axis and 25% for the long axis. It could be seen from the experimental results that the proportion of long and short axes decreased rapidly with the increase in the impact speed. Thus, there was a great difference between the three experimental points with speeds greater than 90 m/s and the theoretical prediction value, as shown in Figure 11. The relative errors of the predictions for both short and long axes increased rapidly with the increase in the impact speed.

In this investigation, it was assumed that the major depressed deformation was an isometric transformation, in order to simplify the model. Based on this method, the original ellipsoid equation could be used to describe the depressed deformation. In fact, according to the comparisons with the experimental results, we found that the theoretical results obtained under this assumption were consistent with the actual measured results in the large deformation and penetration analyses. In practical engineering applications, the large deformation and penetration velocity of ellipsoidal thin shells are key factors that should be considered. Therefore, the theoretical results obtained using this displacement model could provide an important reference for practical applications. The proposed theoretical methodology provided a very efficient model for predicting the general deformation characteristics with an acceptable accuracy. Although not all accurate details of deformation were involved in the theoretical predictions, it was much faster than numerical simulation and more economic than experiments. In addition, it was easy to obtain the influences of various parameters of the shell on the resistance to impact load. This advantage was applied in the following analyses and discussion for the influence of the curvature distribution on the deformation of the impacted ellipsoidal shell.

### 4.2. Impact Resistance Affected by Axial Ratio of Ellipsoidal Shell

Ellipsoidal shells could provide different curvature distributions by changing the triaxial ratio. In order to explore the influence of curvature distribution on the impact resistance of thin curved shells, the dynamic responses of ellipsoidal shells were theoretically analyzed for shells with different triaxial ratios, starting with the spherical shells with an initial triaxial ratio of a:b:c=1:1:1. The axial length of *b* was fixed to 200 mm, and the impact direction was set to the *c* axis, respectively. We stretched or compressed their transverse or longitudinal dimensions to investigate the corresponding change in the impact resistance of the ellipsoidal shells.

**Figure 12:**
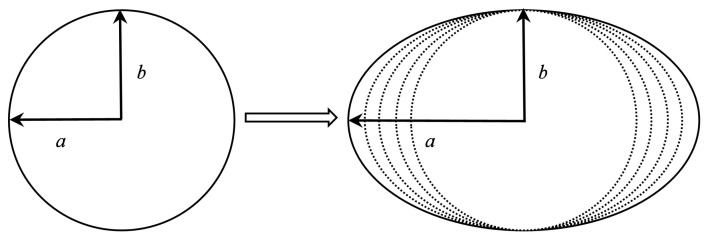
Schematic of shells with different ratios between the *a* and *b* axes.

**Figure 13:**
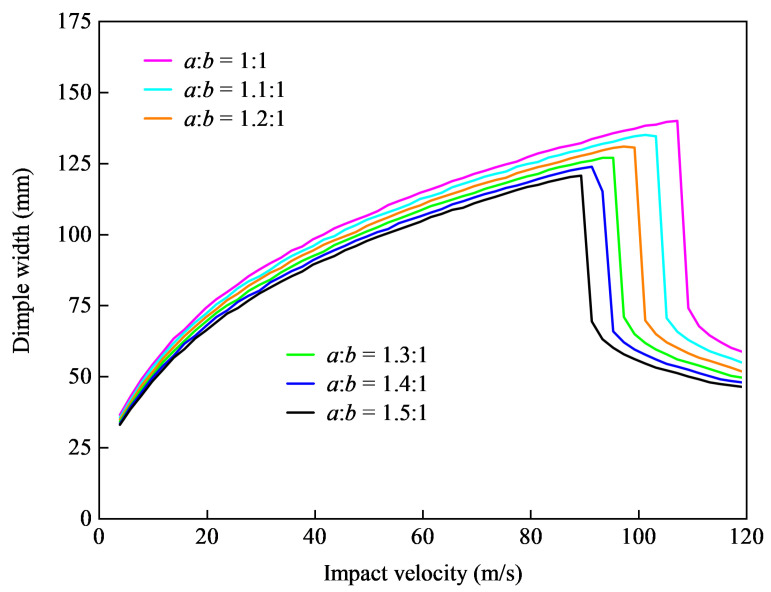
Influence of the ratio between the *a* and *b* axes on the dimple depth, generated by projectiles impacting at different velocities.

We gradually increased the ratio of a:b from 1:1 to 3:2, indicating the stretching of the transverse dimensions of the shell. The specific form of this change is shown in Figure 12. The impact resistance of the shell was measured based on the dimple width in the direction of axis *b*, and the impact speed and dimple width diagram are shown in Figure 13.

The results shown in Figure 13 indicated that with the transverse stretching of the shell, the penetration speed gradually decreased, and the dimple width of the shell gradually decreased under the same impact speed. This showed that the deformation resistance capability could be improved via transverse stretching of the size of the ellipsoidal shell, but the corresponding penetration resistance capability would be reduced.

**Figure 14:**
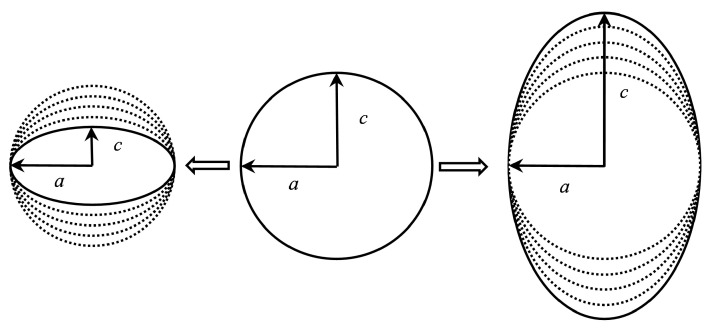
Schematic of shells with different ratios between the *a* and *c* axes.

In order to explore the influence of the tension or compression of the axis size in the impact direction of the ellipsoidal shell on its deformation and penetration, a:c was reduced from 1:1 to 2:3 and increased to 3:2, respectively, to represent its longitudinal tension and compression. The specific form of this change is shown in Figure 14.

**Figure 15:**
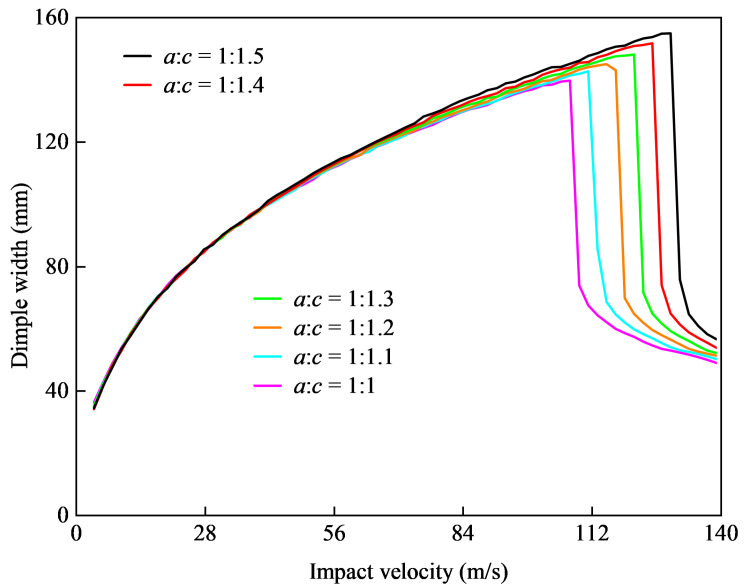
Dimple width changes with the increase in shell depth with a constant length of the *a* axis.

**Figure 16:**
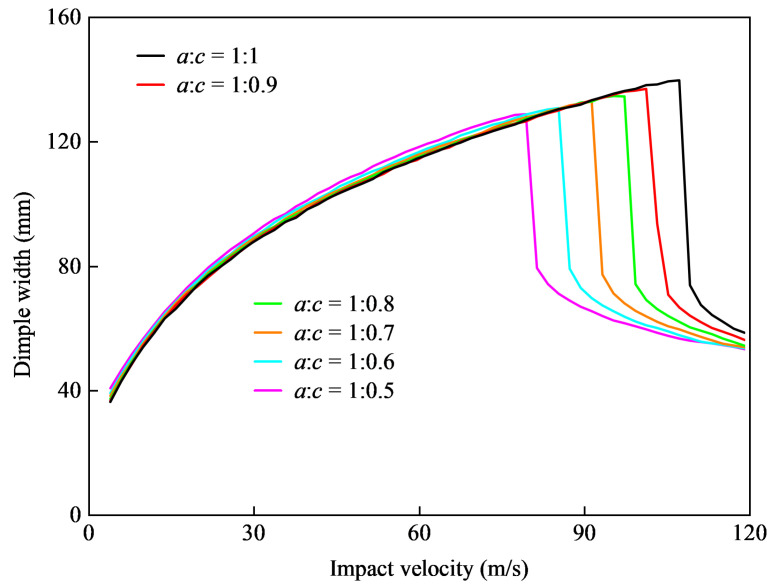
Dimple width changes with a decrease in shell depth with a constant length of the *a* axis.

The impact resistance of the shell was measured based on the dimple width in the direction of axis *a*, and diagrams of the impact speed and dimple width are shown in Figure 15 and Figure 16.

The results shown in Figure 15 and Figure 16 indicated that the longitudinal compression and tension of the initial spherical shell could be attributed to the longitudinal tension of the ellipsoidal shell, because its influence on the shell was consistent. The tension or compression of the axis size in the impact direction had an evident influence on the penetration velocity, rather than on the depressed deformation. This indicated that longitudinal tension for ellipsoidal shells could improve their penetration resistance capability, but had little effect on their capability for deformation resistance.

When the shell was stretched laterally, the integral area of kinetic energy and generalized force was increased at the same time. The membrane force in the edge area provided the major part of the generalized force. The transverse stretching of the shell had a great impact on the generalized force in the edge area, resulting in a reduction of the final depressed deformation. When the shell was stretched longitudinally, it had little effect on the integral region and then had little effect on the kinetic energy term and the generalized force term in the governing equation. Therefore, the longitudinal stretching had little effect on the shell deformation.

**Figure 17:**
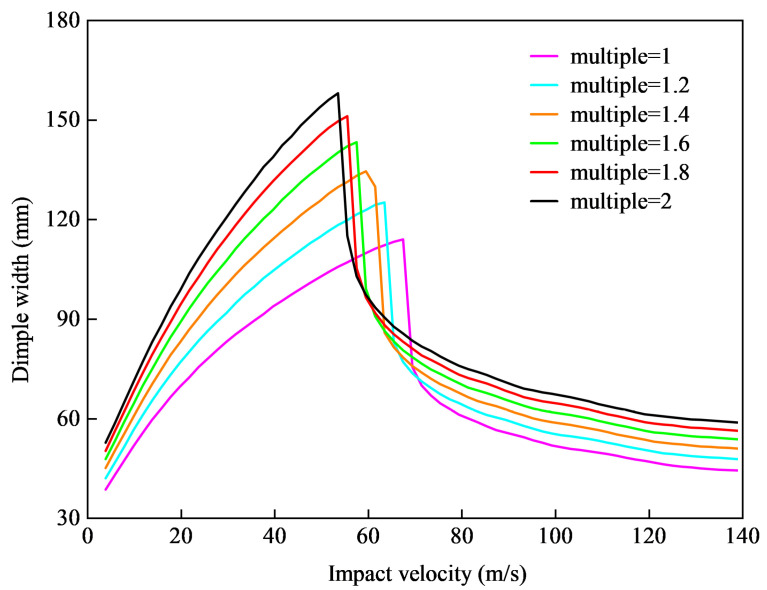
Change in deformation parameters with scaling of the ellipsoidal shell.

In the design process of thin curved shells, not only should one consider the triaxial ratio, but one should also consider the influence of its scaling on its performance. Scaling meant that the ratio between all axes was maintained and the lengths of axes were changed. Therefore, the following analysis was conducted to examine the impact resistance capability affected by the scaling of ellipsoidal shell size. We gradually enlarged the size of the ellipsoidal shell to two times that used in the experiment, measured the impact resistance of the shell according to the short semi-axis length of the dimple area, and constructed a diagram of the impact velocity and dimple width, as shown in Figure 17.

The results shown in Figure 17 indicated that with the enlargement of the overall size of the ellipsoidal shell, the penetration velocity decreased gradually, and the depressed deformation increased gradually under the same impact velocity. This indicated that the size of the ellipsoidal shell had an impact on its impact resistance capability. Specifically, when the triaxial ratio of the ellipsoidal shell remained unchanged, increasing the overall size of the ellipsoidal shell could reduce its deformation and penetration resistance capabilities.

Based on the relationship between the impact velocity and dimple width, it could be observed that the penetration of the shell was induced by the sudden drop in the dimple width, which was the result of the generalized force in the shear region. Region 1 and Region 2 had generalized forces and generalized forces caused by the shear region, respectively. These were a pair of reaction forces, required to prevent the development of the depression in Region 1 and to drive the expansion of the depression in Region 2. When the calculation process indicated that penetration occurred, these two generalized forces failed, resulting in the slow expansion of Region 2, which was manifested in the reduction of the penetration speed. To sum up, the transverse compression, longitudinal tension, and overall reduction in shell size could increase its penetration speed, because these dimensional changes increased the action time of the shear generalized force, which helped the dimple deformation expand.

## 5. Conclusions

In this work, an experimental study of thin-walled metal ellipsoidal shells under the impact of cylindrical projectiles was carried out. Based on the experiments, the final deformation results of ellipsoidal shells under the impact of cylindrical projectiles with different velocities and the key parameters such as the depression depth and width were obtained. At the same time, the dynamic deformation process of ellipsoidal shells was obtained using 3D-DIC technology. The rationality of the use of an isometric transformation in the large deformation region was verified through experiments, and then, a theoretical model based on isometric transformation and the Lagrange principle was established. The theoretical results were in good agreement with the experimental results in the analysis of the large deformation and penetration velocity, which validated the theoretical model established in this paper. Finally, the triaxial dimensions of the ellipsoidal shell in the theoretical model were changed to investigate the influence of the curvature distribution on the impact resistance of the shell. The main conclusions of the experiment can be listed as follows:

(1) The depressed deformation of an ellipsoidal shell under an impact load is elliptical, and the proportions of the long and short axes of the elliptical dimple boundary are close to those of the initial shell.
(2) The isometric transformation was in good agreement with the actual deformation of an ellipsoidal shell in the large deformation region.
(3) Enlarging the axial length of an ellipsoidal shell in the impact direction could improve its resistance to penetration.
(4) Stretching the axial length of an ellipsoidal shell perpendicular to the impact direction could improve its deformation resistance, but this would reduce its resistance to penetration.
(5) Keeping the triaxial ratio unchanged and appropriately reducing the size of an ellipsoidal shell could improve its resistance to deformation and penetration at the same time.

## Figures and Tables

**Table 1:** Geometric and material parameters of thin-walled metal ellipsoidal shells.

Parameter	Value
$L_{1}\left(\mathrm{mm}\right)$	240
$L_{2}\left(\mathrm{mm}\right)$	160
$L_{3}\left(\mathrm{mm}\right)$	40
Elastic modulus E(GPa)	69
Mass density $\rho (\mathrm{kg}\cdot m^{-3})$	2900
Poisson’s ratio ν	0.3
Yield strength $\sigma_{0}\left(\mathrm{GPa}\right)$	0.12

**Table 2:** Deformation modes of impacted ellipsoidal shells.

Case No.	$v_{0}$ (m/s)	Modes	Case No.	$v_{0}$ (m/s)	Modes
1	25.69	II	6	66.32	III
2	36.23	III	7	72.98	II
3	47.24	III	8	92.34	II
4	52.70	IV	9	104.94	I
5	60.78	IV	10	118.97	I

## Data Availability

Not applicable.

## Abbreviations

The following abbreviations are used in this manuscript:

3D	three-dimensional
DIC	digital image correlation

## Appendix A

The shell discussed here is a shallow shell, and the displacement along the direction of impact is approximated as the displacement along the normal direction. The Lame coefficients and curvatures are all represented by local curvilinear coordinates (ξ,η,ζ). It is easy to obtain curvilinear coordinates for axial symmetric rotation shells such as spherical shells and rotation ellipsoidal shells. However, it is difficult to obtain the orthogonal curvilinear coordinate system of an arbitrary ellipsoidal shell. Therefore, a description method of an orthogonal curvilinear coordinate system provided using the methods of the theory of fields was adopted here, that is an orthogonal curvilinear coordinate system composed of a series of an ellipsoid, a univalent hyperboloid, and a bivalent double surface. Then, the coordinates (x,y,z) in the global system can be represented by curvilinear coordinates (ξ,η,ζ). The corresponding base vector is presented in Equation (A1) from base vectors $\left(\vec{i},\vec{j},\vec{k}\right)$ in global coordinates.

(A1) $${\vec{g}}_{i}=\frac{\partial x}{\partial \xi }\vec{i}+\frac{\partial y}{\partial \eta }\vec{j}+\frac{\partial z}{\partial \zeta }\vec{k}$$

Curvilinear coordinates (ξ,η,ζ) were constructed using two hyperbolic surfaces described by ξ and η and one ellipsoidal surface described by ζ. For a given ellipsoidal shell, ζ=0, and the curvatures are governed by ξ and η. The transformation between global coordinates and orthogonal curvilinear coordinates is given as follows [29]:

(A2) $$\begin{array}{cc} x^{2} & =\frac{\left(\xi +a^{2}\right)\left(\eta +a^{2}\right)\left(\zeta +a^{2}\right)}{\left(b^{2}-a^{2}\right)\left(c^{2}-a^{2}\right)}\\ y^{2} & =\frac{\left(\xi +b^{2}\right)\left(\eta +b^{2}\right)\left(\zeta +b^{2}\right)}{\left(a^{2}-b^{2}\right)\left(c^{2}-b^{2}\right)}\\ z^{2} & =\frac{\left(\xi +c^{2}\right)\left(\eta +c^{2}\right)\left(\zeta +c^{2}\right)}{\left(a^{2}-c^{2}\right)\left(b^{2}-c^{2}\right)} \end{array}$$

Lame coefficients are represented as $A_{i}=\sqrt{{\vec{g}}_{i}{\vec{g}}_{i}}\left(i=1,2,3\right)$. This method cannot be reduced to the case of an axisymmetric rotating shell, and if the same method was used to construct the curvilinear coordinates of an axisymmetric rotating shell, the obtained curvilinear coordinates would not be orthogonal. Therefore, this method was only available for the description of ellipsoidal coordinates with unequal triaxial lengths.

Because Region 2 was an isometric transformation of the original shell surface, there was no membrane strain in Region 2. Therefore, the rest membrane strain occurred in Region 3. Based on the experimental analyses, the dimple boundary was almost an ellipse with the same axial ratio as the original shell. This meant that the dimple boundary was in an iso-surface of *z*. Thus, the membrane strain was considered along the normal direction of the dimple boundary, and there was no circular strain in Region 3. Using von Karman large deformation theory along the normal direction of the dimple boundary, the membrane strain was obtained similarly to the spherical shell discussed in [16]. The ellipsoidal equation was rewritten as $x^{\prime }{}^{2}+y^{\prime }{}^{2}+z^{\prime }{}^{2}=1$, where $x=ax^{\prime },y=by^{\prime },z=cz^{\prime }$. The normal direction in the iso-surface along the *z* direction satisfied the following relation for point $\left(x^{\prime },y^{\prime }\right)$.

(A3) $$dx^{\prime }\frac{y^{\prime }}{b^{2}}=dy^{\prime }\frac{x^{\prime }}{a^{2}}$$

The infinitesimal length *d* along the normal direction is represented as follows.

(A4) $$d=\sqrt{dx^{2}+dy^{2}}=\sqrt{a^{2}dx^{\prime 2}+b^{2}dy^{\prime 2}}=a\sqrt{1+\frac{a^{2}y^{\prime 2}}{b^{2}x^{\prime 2}}}dx^{\prime }$$

The differential of the ellipsoidal equation gave the following equation for two points $\left(x^{\prime },y^{\prime },z^{\prime }\right)$ and $\left(x^{\prime }+dx^{\prime },y^{\prime }+dy^{\prime },z^{\prime }-dz^{\prime }\right)$.

(A5) $$\left(2x^{\prime }+dx^{\prime }\right)dx^{\prime }+\left(2y^{\prime }+dy^{\prime }\right)dy^{\prime }=\left(2z^{\prime }-dz^{\prime }\right)dz^{\prime }$$

The normal infinitesimal length could be expressed by Equation (A6), based on Equations (A3) and (A5).

(A6) $$\begin{array}{cc} d & =ax^{\prime }{\left(1+\frac{a^{2}y^{\prime 2}}{b^{2}x^{\prime 2}}\right)}^{\frac{3}{2}}\frac{\sqrt{1+\frac{\left(1+\frac{a^{4}y^{\prime 2}}{b^{4}x^{\prime 2}}\right)\frac{k}{x^{\prime 2}}}{{\left(1+\frac{a^{2}y^{\prime 2}}{b^{2}x^{\prime 2}}\right)}^{2}}}-1}{\left(1+\frac{a^{4}y^{\prime 2}}{b^{4}x^{\prime 2}}\right)}\\ k & =2z^{\prime }dz^{\prime }-dz^{\prime 2} \end{array}$$

where *k* is infinite. Therefore, Equation (A6) could be simplified as follows.

(A7) $$\begin{array}{cc} d & =\frac{1}{2}a{\left(1+\frac{a^{2}y^{\prime 2}}{b^{2}x^{\prime 2}}\right)}^{-\frac{1}{2}}\frac{k}{x^{\prime }}=\frac{1}{2}a{\left(x^{\prime 2}+my^{\prime 2}\right)}^{-\frac{1}{2}}k\\ k & =2z^{\prime }dz^{\prime }-dz^{\prime }{}^{2}≊2z^{\prime }dz^{\prime } \end{array}$$

where $m=\frac{a^{2}}{b^{2}}$. The infinitesimal length considering dz in space before deformation is expressed as follows.

(A8) $$l_{0}=\sqrt{d^{2}+dz^{2}}=\sqrt{c^{2}+a^{2}\frac{z^{\prime }{}^{2}}{G}}dz^{\prime }$$

The corresponding length after deformation is expressed as follows.

(A9) $$\begin{array}{cc} l_{1} & =\sqrt{d^{2}+{\left(dz-w(z)+w(z-dz)\right)}^{2}}=\sqrt{c^{2}{\left(1-\frac{dw}{dz}\right)}^{2}+a^{2}\frac{z^{\prime }{}^{2}}{G}}dz^{\prime }\\ G & =x^{\prime }{}^{2}+my^{\prime }{}^{2}=\left(1-z^{\prime 2}\right)\left[\frac{a^{2}}{b^{2}}\sin^{2}\theta +\cos^{2}\theta \right] \end{array}$$

The displacement distribution in Region 3 is given in Equation (1), which yields the displacement differential with respect to *z* as $\frac{dw}{dz}=2\frac{z-z_{f}+z_{h}}{z_{h}}$. The strain ϵ in the radial normal direction is given as follows.

(A10) $$\begin{array}{cc} \epsilon & =\frac{l_{1}-l_{0}}{l_{0}}=\sqrt{1+4\frac{\frac{{\left(z-z_{f}\right)}^{2}}{z_{h}^{2}}+\frac{\left(z-z_{f}\right)}{z_{h}}}{1+\frac{a^{2}z^{\prime 2}}{c^{2}G}}}-1=\sqrt{1+4p}-1\\ p & =\frac{\frac{{\left(z-z_{f}\right)}^{2}}{z_{h}^{2}}+\frac{\left(z-z_{f}\right)}{z_{h}}}{1+\frac{a^{2}z^{\prime 2}}{c^{2}G}}=\frac{{\left(t+\frac{1}{2}\right)}^{2}-\frac{1}{4}}{1+\frac{a^{2}z^{\prime 2}}{c^{2}G}};\;\;t=\frac{\left(z-z_{f}\right)}{z_{h}} \end{array}$$

where t∈[−1,0], which makes |p|<0.25. Thus, the strain shown in Equation (A10) could be simplified as follows.

(A11) $$\epsilon ≊2p=2\frac{\frac{{\left(z^{\prime }-z_{f}^{\prime }\right)}^{2}}{z_{h}^{\prime }{}^{2}}+\frac{\left(z^{\prime }-z_{f}^{\prime }\right)}{z_{h}^{\prime }}}{1+\frac{a^{2}z^{\prime }{}^{2}}{c^{2}G}}=\frac{2\left(z^{\prime }-z_{f}^{\prime }\right)\left(z^{\prime }-z_{f}^{\prime }+z_{h}^{\prime }\right)c^{2}G}{z_{h}^{\prime }{}^{2}\left(c^{2}G+a^{2}z^{\prime }{}^{2}\right)}$$

where $x^{\prime }=\sqrt{1-z^{\prime }{}^{2}}\cos\theta$ and $y^{\prime }=\sqrt{1-z^{\prime }{}^{2}}\sin\theta$ represent the ellipsoidal isosurface along the *z* direction in cylindrical coordinates.

The final expression of the strain distributed in Region 3 is described by Equation (A11). It was applied in the expression of general force.
